# Association between elevated glycosylated hemoglobin and cognitive impairment in older Korean adults: 2009–2010 Ansan cohort of the Korean genome and epidemiology study

**DOI:** 10.3389/fpubh.2024.1417236

**Published:** 2024-11-11

**Authors:** Jung Sook Kim, Byung Chul Chun, Kyoungho Lee

**Affiliations:** ^1^Division of Population Health Research, Department of Precision Medicine, Korea National Institute of Health, Cheongju-si, Chungcheongbuk-do, Republic of Korea; ^2^Department of Preventive Medicine, Korea University College of Medicine, Seoul, Republic of Korea

**Keywords:** older people, Korean version of the mini-mental state examination, Korean version of the Montreal cognitive assessment, cognitive impairment, glycosylated hemoglobin

## Abstract

**Objective:**

Cohort studies on the risk of cognitive impairment in the older population of S. Korea based on glycosylated hemoglobin (HbA1c) levels are exceedingly rare. This study aimed to analyze the association between HbA1c levels and cognitive impairment in older Korean adults without dementia.

**Methods:**

We conducted a cross-sectional study using data from a community-based Ansan cohort (2009–2010), which was part of the Korea Genome Epidemiology Study. The study included 853 cohort participants aged ≥59 years living in Ansan city. Cognitive function was evaluated using the Korean version of the Mini-Mental State Examination (MMSE) and Montreal Cognitive Assessment (MoCA). The MMSE and MoCA scores were categorized into normal cognition (≥24 and ≥ 23, respectively) and cognitive impairment (≤23 and ≤ 22, respectively). Multiple logistic regression analysis was used to estimate the association between HbA1c levels and cognitive impairment, with adjustments for covariates.

**Results:**

The mean age of the participants was 66 years, and 433 (50.8%) were female. Cognitive impairment was observed in 12.5 and 44.3% of participants, based on the MMSE and MoCA, respectively. Regarding the MMSE scores, HbA1c level was a risk factor for cognitive impairment in women. Compared to normal HbA1c (≤5.6%) levels, adjusted odds ratios of MMSE decline for HbA1c 5.7–6.4% and HbA1c ≥6.5% were high: 2.16 (95% confidence interval [CI] 1.04–4.49) and 2.96 (95% CI, 1.04–8.39), respectively.

**Conclusion:**

By improving glycemic control, the risk of cognitive impairment in the older population can be reduced. Further research on the role of sex differences in cognitive impairment is needed.

## 1 Introduction

As the global population ages, the prevalence of chronic diseases such as dementia and diabetes in older adults is increasing. The early detection of cognitive dysfunction is important to prevent dementia, which makes independent living difficult, and for promoting healthy aging in the older adults. Cognitive decline is a major cause of disability and contributes to increased mortality (1). However, as cognitive decline encompasses a long process prior to the onset of dementia, identifying risk factors may help to screen individuals who could benefit from early intervention (2). Moreover, cognitive decline is known to occur mainly in patients with diabetes-related complications, especially microvascular or macrovascular complications (3).

Glycosylated hemoglobin (HbA1c) is an important biomarker of long-term glycemic control, representing the average blood glucose level over the past 2 to 3 months. It is also used as a diagnostic indicator for diabetes and assessing risk of complications (4). Most studies have evaluated the relationship between glycemic control and cognitive function based on diabetes status (5–9). However, the biological mechanisms underlying the relationship between diabetes and cognitive decline remain unclear (5). A previous study (2) showed a linear correlation between circulating HbA1c levels and cognitive decline, regardless of diabetes status. Moreover, in a cohort of healthy older adults without dementia or diabetes, HbA1c showed a strong correlation with memory performance. In addition, chronically high blood glucose levels had a negative impact on cognition owing to structural changes in learning-related brain areas (10). Additionally, elevated HbA1c has been associated with an increased risk of all-cause dementia and Alzheimer’s disease dementia in older individuals. HbA1c levels have been found to be associated with risk of mild cognitive impairment or dementia in postmenopausal older women, mainly those without diabetes (11). Although HbA1c is known as a long-term glycemic control management indicator for diabetes, the role of HbA1c as a risk factor for cognitive impairment remains unclear.

In a previous study (12) in South Korea, chronic hyperglycemia caused atrophic changes in the frontal lobe and cerebellum as well as structural changes in the brain, which led to cognitive decline in older adults. In a previous study that investigated the risk factors and patterns of cognitive decline in community-dwelling older Korean adults (age ≥ 65 years), older age, female sex, absence of religious beliefs, residence in a small city, high number of chronic diseases, depression, lack of exercise, and alcohol consumption were found to be the risk factors associated with cognitive decline (13). Moreover, a validation study of the MoCA (Montreal Cognitive Assessment)–MMSE (Mini-Mental State Examination) conversion scales for patients with cognitive impairment in S. Korea was also conducted (14).

Studying risk factors for cognitive impairment in older adults using community-based cohorts is essential for promoting healthy aging in older adults without dementia. However, the role of HbA1c as a risk factor for cognitive impairment in S. Korea remains unclear. Additionally, few cohort studies have investigated the association between HbA1c levels and the risk of cognitive impairment in the older population. Therefore, we conducted a cross-sectional study to determine the level of HbA1c in the study population and to assess the risk of cognitive impairment according to the level of HbA1c. We hypothesized that a difference in the risk of cognitive impairment in the older population would be observed when HbA1c levels are above the normal range. Therefore, this study investigated the relationship between cognitive impairment and HbA1c levels, serving as a blood-based biomarker, in older Korean adults without dementia using the Korean version of the representative cognitive function assessment tool (K-MMSE, K-MoCA).

## 2 Materials and methods

### 2.1 Study participants

The KoGES, an ongoing longitudinal prospective cohort study, was initiated by the Korean government [National Research Institute of Health (NIH)] in 2001 to identify genetic and environmental factors of chronic diseases prevalent among Koreans. The characteristic details of the KoGES and core variables collected have been described in a previous study (15).

This study used data from the community-based Ansan cohort, which is part of the representative cohorts in the KoGES. The baseline survey of the Ansan cohort was conducted from 2001 to 2002 by sampling a total of 5,012 adults from the target population of men and women aged 40 to 69 years living in Ansan City in 2000. Since 2001, follow up with study participants has been conducted biennially.

We conducted a cross-sectional study on the association between HbA1c and cognitive impairment using data collected from the fifth wave of the Ansan cohort study (2009–2010). Cohort participants engaged in a battery of comprehensive tests, including questionnaires (socio-demographic characteristics, lifestyle [smoking, drinking, physical activity (16) and diet (17)], disease history), anthropometric measures, clinical examination (blood and urine test) and cognitive function tests (MMSE and MoCA: age ≥ 60 years) from researchers who have received professional training on a standardized protocol (18). Complete MMSE and MoCA assessments were required for inclusion in the present analysis.

**Figure 1:**
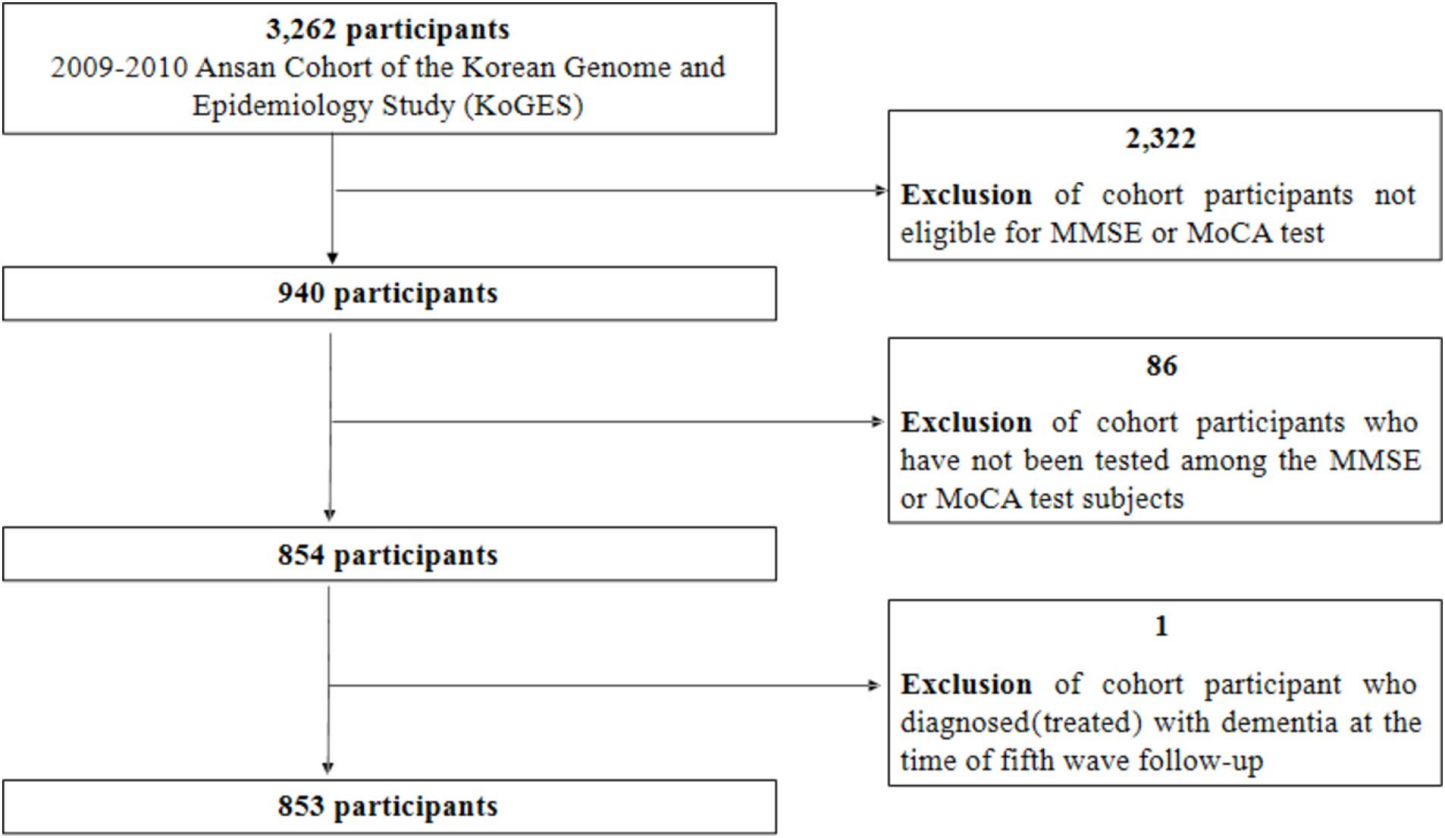
Selection of study participants. MMSE, Mini-Mental State Examination; MoCA, Montreal Cognitive Assessment.

Among the 3,262 participants from the cohort, 2,322 participants under 60 years of age were excluded because they were ineligible for two cognitive function tests (MMSE or MoCA). Additionally, among those eligible for the two cognitive function tests, 86 participants whose cognitive function was not assessed were excluded. Finally, among the 854 remaining participants, 853 were selected as the final research participants after excluding one person who was receiving dementia treatment (frequency of dementia treatment: 6 times/year; Figure 1). Among the total 853 participants, 849 were tested for MMSE and four were excluded due to missing values in the sum of MMSE scores. There were no missing data in the seven domains of the 849 MMSE tests. Of the 853 participants, 846 were tested for MoCA, excluding seven people with missing values in the sum of MoCA scores. Among these 846 participants, one point was added to the MoCA score for those who reported education level was elementary school or lower. Seven participants with missing education levels were also excluded, resulting in a final total of 839 participants for the MoCA test. Cognitive decline was reported to be significantly greater in adults with diabetes compared to adults without diabetes in a community-based population (19). In this study, diabetes was also associated with glycated hemoglobin (χ^2^ = 1196.06, *p* < 0.0001) and was judged to be a confounding variable affecting MMSE decline (χ^2^ = 10.39, *p* = 0.0055) and MoCA decline (χ^2^ = 6.31, *p* = 0.0427), so it was excluded from the analysis of this study.

### 2.2 Clinical laboratory information and physical measurement

Sociodemographic variables (sex, age, and education level), lifestyle habits (drinking and smoking history), anthropometric measurements (body mass index, systolic blood pressure, diastolic blood pressure, and muscle mass), and subjective health status (bad [including very bad], fair, and good [including very good]) that can affect cognitive function were investigated. Clinical information (HbA1c, fasting blood glucose, insulin, hemoglobin, total cholesterol, high-density lipoprotein cholesterol, triglycerides, and homocysteine) obtained from blood biochemical tests of the participants was included in the analysis. The average degree of glycemic control in the participants over the past 3 months was evaluated using the HbA1c level, which was expressed as a percentage. HbA1c level, which was investigated as a continuous variable, was categorized into three groups using the criteria of previous studies (20–22): HbA1c ≤5.6% (normal); HbA1c: 5.7–6.4% (prediabetes); HbA1c ≥6.5% (diabetes). In order to identify the risk of cognitive impairment in HbA1c 5.7–6.4% and HbA1c ≥6.5% group, HbA1c ≤5.6% group was set as the reference group.

### 2.3 Assessment of cognitive function

The MMSE has long been the most widely used screening test for dementia in clinical settings (23, 24). The MoCA was developed as a screening tool for detecting mild cognitive impairment. As the MoCA includes more robust measures of visuospatial and executive functions, it has excellent sensitivity for mild cognitive impairment detection and can be used in clinical settings (25). In this study, we aimed to increase the validity of the results by examining whether cognitive impairment measured by MMSE and MoCA is related to HbA1c. The results of the validity study on the K-MMSE in dementia patients, which was verified in Korean, showed that the sensitivity of the K-MMSE for dementia diagnosis was 0.70–0.83 (26). In a study to verify the validity of K-MoCA as a screening test for vascular cognitive impairment (VCI) in patients with stroke and normal older adult subjects, which was also validated in Korean, the receiver operating curve (ROC) analysis results showed that K-MoCA could discriminate well between the VCI group and the normal group (area under the ROC = 0.80, *p* < 0.001) (27). To confirm the strength and direction of the relationship between the MMSE and MoCA tests, a Pearson correlation analysis was performed, and the correlation coefficient was 0.657 (*p* < 0.0001).

The cognitive function of the study participants was evaluated using the MMSE and MoCA tests, with scores ranging up to 30. Higher scores indicated better cognitive function. The MMSE and MoCA tests each consisted of seven domains, and the average and standard deviation for each domain are shown in Supplementary Appendix 1 Table 1. Education level can affect the performance of MoCA and MMSE tests (28). MMSE is not suitable for detecting mild cognitive impairment (MCI) in people with a high level of education (29), and MoCA was developed to screen for MCI by including an extended assessment of executive and visuospatial functions (30). In prior domestic studies, K-MoCA added one point to the scores of patients with 6 years or less of education (14, 31–33), and adjusted scores to validate the MoCA-MMSE conversion scale for patients with cognitive impairment. In a study overseas (25), it was suggested that those with 12 years or less of education receive additional points on the total MoCA score. Thus, in the present study, the MoCA scores were adjusted by adding one point to scores of participants with an education level ≤ 6 years.

### 2.4 Definition of cognitive impairment

Among the study participants, 849 completed the Korean version of the MMSE (MMSE group). We followed the conventional classification criteria for cognitive function, categorizing K-MMSE scores as severe cognitive impairment (SCI, K-MMSE ≤17), mild cognitive impairment (MCI, 18 ≤ K-MMSE ≤23), and normal cognitive function (K-MMSE ≥24) (26, 34). In this study, frequency analysis of the total score of the MMSE test revealed that severe cognitive impairment was present in 1.4% of participants, and mild cognitive impairment was present among 11.1% of participants. The two groups were combined and named the “cognitive impairment group.” Normal cognitive function was present among 87.5% of participants, who were labeled the “normal cognition group.” Thus, based on the MMSE cut-off score of 24 points, participants were classified into the “normal cognition group” (≥24 points) and “cognitive impairment group” (≤23 points).

A total of 839 participants completed the Korean version of the MoCA (MoCA group). Based on the MoCA cut-off score of 23 points (33), participants were classified into the “normal cognition group” (≥23 points) and “cognitive impairment group” (≤22 points).

### 2.5 Statistical analysis

Continuous variables are presented as mean ± standard deviation, while categorical variables are expressed as number (%). However, continuous variables that did not follow a normal distribution were represented as medians. We analyzed the characteristics of the study participants based on the three subgroups of HbA1c (≤5.6%, 5.7–6.4%, ≥6.5%). We also compared these characteristics between the normal cognition and cognitive impairment groups, as assessed by the MMSE and MoCA. Categorical variables were analyzed using the chi-square test, while continuous variables were analyzed using a general linear model. After checking for mean differences using the general linear model test, groups with different means were identified via multiple comparisons with Bonferroni correction.

In the multiple logistic regression analysis, the risk of cognitive impairment, as assessed by MMSE and MoCA, was used as the dependent variable. Variables with a *p*-value of ≤0.2 in the univariate analysis results, along with clinical information [lipids (35, 36), homocysteine (37, 38)] known to be related to cognitive decline from previous studies were included in the regression model as independent variables. All independent variables were simultaneously entered into the regression model. The goodness of fit of the model was assessed using the Hosmer–Lemeshow test. After adjusting for risk factors affecting cognitive function, multiple logistic regression analysis was performed to calculate the risk of cognitive impairment (odds ratios and 95% confidence intervals [CIs]) when the HbA1c level was above the normal range. Statistical analyses were conducted using SAS version 9.4 (SAS Institute, Cary, NC, United States). Statistical significance was set at *p* < 0.05 (two-tailed).

## 3 Results

The demographic and clinical characteristics of the study participants are shown in Table 1. Among the 853 participants (mean age, 66.4 ± 4.7 years), 420 (49.2%) were male and 433 (50.8%) were female. Among the 846 people who responded to questions about their education level, those with elementary school or lower were the most common at 282 (33.3%), followed by high school at 253 (29.9%), middle school at 167 (19.8%), and junior college or higher at 144 (17.0%). For the MMSE, the scaled score by domain was the lowest in the recall domain at 49.7%. For the MoCA, the abstraction score was the lowest at 31.5% (Supplementary Appendix 1 Table 1). The mean MMSE and MoCA scores were 26.3 ± 2.8 and 22.4 ± 4.1, with median values of 27.0 and 23.0, respectively (Supplementary Appendix 2 Table 2). The minimum MMSE score was 13 points, the maximum 30 points, and the range was 17. The minimum MoCA score was six points, the maximum 30 points, and the range was 24.

**Table 1:** Clinical characteristics and cognitive function of study participants.

Variable	Category	N	n (%)	Mean ± SD
Sex	Male	853	420 (49.2)	
	Female		433 (50.8)	
Age (years, continuous)		853		66.4 ± 4.7
Age (years, category)	59–64	853	360 (42.2)	
	65–69		255 (29.9)	
	≥70		238 (27.9)	
Education level	≤Elementary school	846	282 (33.3)	
	≥Middle school		564 (66.7)	
Alcohol use status	Never alcohol use	853	476 (55.8)	
	Former alcohol use		34 (4.0)	
	Current alcohol use		343 (40.2)	
Smoking status	Never smoker	853	517 (60.6)	
	Former smoker		262 (30.7)	
	Current smoker		74 (8.7)	
BMI (kg/m^2^)		852		24.8 ± 3.0
SBP (mmHg)		846		120.0 ± 16.2
DBP (mmHg)		846		74.6 ± 9.0
Muscle mass (kg)		852		42.7 ± 7.5
HbA1c (%, continuous)		853		5.9 ± 0.9
HbA1c (%, category)		853		
	≤5.6		341 (40.0)	
	5.7–6.4		371 (43.5)	
	≥6.5		141 (16.5)	
FBG (mg/dL)		853		103.5 ± 31.1
Insulin (μIU/mL)		853		10.6 ± 16.4
Hb (g/dL)		853		13.7 ± 1.3
TC (mg/dL)		853		197.6 ± 38.1
HDL-C (mg/dL)		853		44.2 ± 10.9
TG (mg/dL)		853		139.3 ± 74.3
Homocysteine (μmol/L)		853		14.8 ± 5.3
Subjective health status	Bad	853	192 (22.5)	
	Fair		361 (42.3)	
	Good		300 (35.2)	
Cognitive function	MMSE score	849		26.3 ± 2.8
	MoCA score	839		22.4 ± 4.1

SD, standard deviation; MMSE, Mini-Mental State Examination; MoCA, Montreal Cognitive Assessment; BMI, body mass index; SBP, systolic blood pressure; DBP, diastolic blood pressure; HbA1c, glycosylated hemoglobin; FBG, fasting blood glucose; Hb, hemoglobin; TC, total cholesterol; HDL-C, high-density lipoprotein cholesterol; TG, triglycerides.

The results of the analysis of the association between participant characteristics and the three categories of HbA1c levels are shown in Table 2. The HbA1c 5.7–6.4% group comprised significantly more women (215; 49.7%) than men (156; 37.2%; *p* = 0.001). The MMSE score was significantly lower in the HbA1c ≥6.5% group (25.7 ± 3.2) than in the HbA1c 5.7–6.4% (26.4 ± 2.6) and HbA1c <5.6% (26.5 ± 2.6) groups (all *p* < 0.05). The MoCA score was also significantly lower in the HbA1c ≥6.5% group (21.3 ± 4.8) than in the HbA1c 5.7–6.4% (22.5 ± 4.0) and HbA1c <5.6% (22.6 ± 3.8) groups (all *p* < 0.01).

**Table 2:** Association between characteristics of study participants and glycosylated hemoglobin level.

Variable	Category	HbA1c (%, category)			Bonferroni	*p*
		≤ 5.6 (*n* = 341, 40.0%)	5.7–6.4 (*n* = 371, 43.5%)	≥ 6.5 (*n* = 141, 16.5%)		
Sex	Male	190 (45.2)	156 (37.2)	74 (17.6)		0.001
	Female	151 (34.9)	215 (49.7)	67 (15.4)		
Age (years, continuous)		66.3 ± 4.6	66.5 ± 4.8	66.5 ± 4.8		0.733
Age (years, category)	59–64	142 (39.4)	158 (43.9)	60 (16.7)		0.377
	65–69	114 (44.7)	102 (40.0)	39 (15.3)		
	≥70	85 (35.7)	111 (46.6)	42 (17.7)		
Education level	≤Elementary school	96 (34.0)	137 (48.6)	49 (17.4)		0.047
	≥Middle school	241 (42.7)	232 (41.1)	91 (16.2)		
Alcohol use status	Never alcohol use	169 (35.5)	223 (46.9)	84 (17.6)		0.028
	Former alcohol use	14 (41.2)	12 (35.3)	8 (23.5)		
	Current alcohol use	158 (46.1)	136 (39.7)	49 (14.2)		
Smoking status	Never smoker	194 (37.5)	241 (46.6)	82 (15.9)		0.243
	Former smoker	116 (44.3)	101 (38.6)	45 (17.1)		
	Current smoker	31 (41.9)	29 (39.2)	14 (18.9)		
BMI (kg/m^2^)		24.2 ± 2.9C	24.9 ± 3.0B	25.7 ± 2.8A	A-B*, A-C***, B-C**	<0.0001
SBP (mmHg)		119.6 ± 16.0	120.1 ± 17.0	120.8 ± 14.5		0.748
DBP (mmHg)		74.5 ± 8.6C	75.3 ± 9.2B	72.7 ± 9.4A	A-B**	0.011
Muscle mass (kg)		43.1 ± 7.1C	41.8 ± 7.7B	44.4 ± 7.4A	A-B**	0.001
HbA1c (%, continuous)		5.3 ± 0.3C	5.9 ± 0.2B	7.4 ± 1.1A	A-B***, A-C***, B-C***	<0.0001
FBG (mg/dL)		92.4 ± 8.1C	98.3 ± 12.8B	144.0 ± 57.4A	A-B***, A-C***, B-C**	<0.0001
Insulin (μIU/mL)		8.0 ± 3.1C	9.7 ± 5.3B	19.0 ± 37.9A	A-B***, A-C***	<0.0001
Hb (g/dL)		13.8 ± 1.4	13.6 ± 1.3	13.6 ± 1.5		0.159
TC (mg/dL)		196.9 ± 34.4C	202.3 ± 40.5B	187.2 ± 38.0A	A-B**, A-C*	0.001
HDL-C (mg/dL)		45.6 ± 11.0C	44.0 ± 11.2 B	41.2 ± 8.9A	A-B*, A-C**	0.001
TG (mg/dL)		122.7 ± 64.3C	146.0 ± 76.7B	161.5 ± 81.7A	A-C***, B-C***	<0.0001
Homocysteine (μmol/L)		14.9 ± 5.1C	14.5 ± 5.9B	15.3 ± 4.3A		0.325
Subjective health status	Bad	63 (32.8)	81 (42.2)	48 (25.0)		0.002
	Fair	159 (44.0)	147 (40.7)	55 (15.3)		
	Good	119 (39.7)	143 (47.7)	38 (12.6)		
Cognitive function	MMSE score	26.5 ± 2.6C	26.4 ± 2.6B	25.7 ± 3.2A	A-B*, A-C*	0.009
	MoCA score	22.6 ± 3.8C	22.5 ± 4.0B	21.3 ± 4.8A	A-B**, A-C**	0.002

Categorical variables were analyzed using the chi-square test, and continuous variables were analyzed using the GLM. Superscript letters (a, b, c) denote the results of the Bonferroni multiple comparisons across groups. Statistically significant p values are indicated as *p < 0.05, **p < 0.01, ***p < 0.0001. MMSE, Mini-Mental State Examination; MoCA, Montreal Cognitive Assessment; BMI, body mass index; SBP, systolic blood pressure; DBP, diastolic blood pressure; HbA1c, glycosylated hemoglobin; FBG, fasting blood glucose; Hb, hemoglobin; TC, total cholesterol; HDL-C, high-density lipoprotein cholesterol; TG, triglycerides.

Table 3 displays the results of the association analysis for factors related to the MMSE and MoCA scores in older adults without dementia; The cognitive impairment group with a score of 23 or lower on the MMSE test included 12.5% of participants, while the cognitive impairment group with a score of 22 or lower on the MoCA test was higher, with 44.3% of participants. Furthermore, the group suspected of cognitive impairment (MMSE ≤23 and MoCA ≤22) comprised significantly more women (17.2 and 48.7%) than men (7.6 and 39.9%; *p* < 0.0001 and *p* = 0.010, respectively). For age category, those over 70 years of age comprised 19.8% of the cognitive impairment group on the MMSE (*p* < 0.0001) and 57.1% of the cognitive impairment group by MoCA results (*p* < 0.0001) compared with the cognitively normal groups. For education, the proportion of cognitively impaired participants was lower for elementary school at 25.5% by MMSE, and higher for elementary school at 61.6% by MoCA, than in the middle school or higher level, respectively (*p* < 0.0001, *p* < 0.0001).

**Table 3:** Association analysis for factors related to MMSE and MoCA scores in study participants.

Variable	Category	MMSE (*n* = 849)		*p*	MoCA (*n* = 839)		*p*
		≤23, *n* = 106 (12.5%)	≥24, *n* = 743 (87.5%)		≤22, *n* = 372 (44.3%)	≥23, *n* = 467 (55.7%)	
Sex	Male	32 (7.6)	387 (92.4)	<0.0001	165 (39.9)	249 (60.1)	0.010
	Female	74 (17.2)	356 (82.8)		207 (48.7)	218 (51.3)	
Age (years, category)	59–64	22 (6.1)	338 (93.9)	<0.0001	120 (33.6)	237 (66.4)	<0.0001
	65–69	37 (14.7)	215 (85.3)		120 (47.8)	131 (52.2)	
	≥70	47 (19.8)	190 (80.2)		132 (57.1)	99 (42.9)	
Education level	≤Elementary school	72 (25.5)	210 (74.5)	<0.0001	172 (61.6)	107 (38.4)	<0.0001
	≥Middle school	33 (5.9)	531 (94.1)		200 (35.7)	360 (64.3)	
Alcohol drinking	Never alcohol drinker	68 (14.4)	406 (85.6)	0.028	212 (45.3)	256 (54.7)	0.087
	Past alcohol drinker	7 (20.6)	27 (79.4)		20 (60.6)	13 (39.4)	
	Current alcohol drinker	31 (9.1)	310 (90.9)		140 (41.4)	198 (58.6)	
Smoking status	Never smoker	80 (15.6)	434 (84.4)	0.001	243 (47.8)	265 (52.2)	0.026
	Past smoker	17 (6.5)	245 (93.5)		97 (37.6)	161 (62.4)	
	Current smoker	9 (12.3)	64 (87.7)		32 (43.8)	41 (56.2)	
Subjective health status	Good	36 (12.0)	263 (88.0)	0.001	110 (37.4)	184 (62.6)	<0.0001
	Fair	32 (8.9)	326 (91.1)		151 (42.5)	204 (57.5)	
	Bad	38 (19.8)	154 (80.2)		111 (58.4)	79 (41.6)	
BMI (kg/m^2^)		25.3 ± 3.2	24.7 ± 2.9	0.030	24.8 ± 3.0	24.7 ± 2.9	0.695
SBP (mmHg)		122.9 ± 17.9	119.6 ± 15.9	0.046	122.0 ± 18.0	118.5 ± 14.5	0.002
DBP (mmHg)		75.5 ± 10.5	74.5 ± 8.8	0.268	75.0 ± 9.6	74.4 ± 8.5	0.311
Muscle mass (kg)		40.1 ± 6.6	43.1 ± 7.5	0.001	41.8 ± 7.4	43.4 ± 7.4	0.002
HbA1c (%, category)	≤5.6	31 (9.1)	308 (90.9)	0.001	143 (42.7)	192 (57.3)	0.086
	5.7–6.4	45 (12.2)	324 (87.8)		156 (42.6)	210 (57.4)	
	≥6.5	30 (21.3)	111 (78.7)		73 (52.9)	65 (47.1)	
HbA1c (%, continuous)		6.2 ± 1.0	5.9 ± 0.8	0.001	6.0 ± 1.0	5.9 ± 0.8	0.034
FBG (mg/dL)		110.8 ± 43.4	102.5 ± 28.9	0.011	105.9 ± 35.4	101.7 ± 27.3	0.053
Insulin (μIU/mL)		13.7 ± 24.8	10.1 ± 14.8	0.036	10.7 ± 14.1	10.5 ± 18.1	0.865
Hb (g/dL)		13.3 ± 1.2	13.7 ± 1.3	0.007	13.6 ± 1.3	13.7 ± 1.3	0.216
TC (mg/dL)		197.8 ± 37.2	197.5 ± 38.3	0.949	196.0 ± 38.0	198.9 ± 38.4	0.277
HDL-C (mg/dL)		43.9 ± 11.4	44.2 ± 10.7	0.789	44.3 ± 11.0	44.1 ± 10.7	0.878
TG (mg/dL)		141.4 ± 70.4	138.4 ± 73.4	0.692	140.6 ± 75.2	137.0 ± 71.6	0.482
Homocysteine (μmol/L)		14.8 ± 4.5	14.8 ± 5.4	0.895	14.8 ± 5.5	14.7 ± 5.1	0.702

Categorical variables were analyzed using the chi-square test. Categorical and continuous variables were analyzed using a general linear model (GLM). MMSE, Mini-Mental State Examination; MoCA, Montreal Cognitive Assessment; BMI, body mass index; SBP, systolic blood pressure; DBP, diastolic blood pressure; HbA1c, glycosylated hemoglobin; FBG, fasting blood glucose; Hb, hemoglobin; TC, total cholesterol; HDL-C, high-density lipoprotein cholesterol; TG, triglycerides.

Continuous HbA1c levels were significantly higher in the MMSE and MoCA groups suspected of cognitive impairment (6.2 ± 1.0 and 6.0 ± 1.0) than in the normal cognition group (5.9 ± 0.8 and 5.9 ± 0.8; *p* = 0.001 and 0.034, respectively). However, when HbA1c was categorized into three groups, in the MMSE cognitive impairment group, the HbA1c ≥6.5% group was the largest, comprising 30 (21.3%) participants. Conversely, in the normal cognition group, the HbA1c ≤5.6% group was the largest, comprising 308 (90.9%) individuals. This difference was significant (*p* = 0.001). However, in the MoCA cognitive impairment group, the HbA1c ≥6.5% group was the largest, comprising 73 (52.9%) individuals. In contrast, in the normal cognition group, the HbA1c 5.7–6.4% group was the largest, comprising 210 (57.4%) participants. However, this discrepancy did not reach statistical significance (*p* = 0.086).

Table 4 presents the results of multivariate logistic regression analysis for factors related to “cognitive impairment,” defined as an MMSE score ≤ 23 or MoCA score ≤ 22. Evaluation of cognitive function through the MMSE and MoCA indicated that the risk of cognitive impairment in adults significantly increased with age and an education level of elementary school or lower. For women with a poor subjective health status, the risk of cognitive impairment was 2.12 (95% CI, 1.02–4.37) and 3.05 (95% CI, 1.73–5.40) times.

**Table 4:** Multiple logistic regression analysis of MMSE and MoCA score decline

Variable		MMSE decline OR (95% CI)			MoCA decline OR (95% CI)		
		Total	Male	Female	Total	Male	Female
Sex (female = 1)		1.36 (0.44, 4.23)	-	-	1.15 (0.57, 2.32)	-	-
Age (years)		1.12 (1.07, 1.19)***	1.16 (1.06, 1.27) **	1.13 (1.06, 1.22) **	1.09 (1.06, 1.13) ***	1.11 (1.05, 1.17) **	1.09 (1.04, 1.15) **
Education level (≤elementary school = 1)		4.19 (2.51, 7.00)***	3.98 (1.58, 10.07) **	5.78 (2.85, 11.69) ***	2.47 (1.77, 3.47) ***	2.46 (1.39, 4.36) **	2.61 (1.69, 4.05) ***
Alcohol use status (ever drinking alcohol = 1)		1.29 (0.73, 2.28)	1.41 (0.55, 3.63)	1.18 (0.55, 2.56)	1.29 (0.90, 1.84)	1.08 (0.67, 1.74)	1.75 (1.00, 3.07)
Smoking status (ever smoker = 1)		0.81 (0.35, 1.90)	1.11 (0.40, 3.07)	0.60 (0.06, 5.74)	0.74 (0.46, 1.18)	0.82 (0.49, 1.39)	0.93 (0.22, 3.85)
BMI (kg/m^2^)		1.01 (0.92, 1.12)	0.83 (0.67, 1.03)	1.04 (0.92, 1.18)	0.97 (0.91, 1.04)	1.00 (0.90, 1.11)	0.96 (0.87, 1.05)
SBP (mmHg)		1.00 (0.98, 1.01)	0.99 (0.96, 1.03)	1.00 (0.98, 1.02)	1.01 (0.99, 1.02)	0.99 (0.97, 1.01)	1.02 (1.00, 1.04)
DBP (mmHg)		1.04 (1.00, 1.08) *	1.07 (1.01, 1.14) *	1.03 (0.98, 1.08)	1.01 (0.99, 1.04)	1.03 (1.00, 1.07)	1.00 (0.96, 1.03)
Muscle mass (kg)		0.99 (0.93, 1.06)	0.99 (0.90, 1.10)	1.03 (0.93, 1.13)	1.00 (0.96, 1.04)	0.99 (0.95, 1.04)	0.99 (0.92, 1.06)
Subjective health status	Good	Reference	Reference	Reference	Reference	Reference	Reference
	Fair	0.75 (0.43, 1.31)	0.54 (0.21, 1.38)	0.88 (0.41, 1.89)	1.41 (1.00, 1.98)	1.32 (0.82, 2.11)	1.45 (0.85, 2.46)
	Bad	1.41 (0.80, 2.50)	0.32 (0.08, 1.24)	2.12 (1.02, 4.37) *	2.27 (1.51, 3.42) ***	1.47 (0.77, 2.84)	3.05 (1.73, 5.40) **
FBG (mg/dL)		1.00 (1.00, 1.01)	1.00 (0.98, 1.01)	1.01 (1.00, 1.02)	1.00 (1.00, 1.01)	1.01 (1.00, 1.02)	1.00 (0.99, 1.01)
Insulin (μIU/mL)		1.00 (0.99, 1.02)	1.00 (0.97, 1.03)	1.00 (0.99, 1.02)	1.00 (0.99, 1.01)	1.00 (0.98, 1.02)	1.00 (0.98, 1.01)
Hb (g/dL)		0.99 (0.79, 1.25)	1.08 (0.75, 1.54)	0.85 (0.60, 1.20)	1.09 (0.94, 1.27)	1.01 (0.83, 1.22)	1.25 (0.96, 1.62)
TC (mg/dL)		1.00 (1.00, 1.01)	0.99 (0.98, 1.01)	1.01 (1.00, 1.02)	1.00 (0.99, 1.00)	1.00 (0.99, 1.01)	1.00 (0.99, 1.00)
HDL-C (mg/dL)		0.99 (0.97, 1.02)	0.93 (0.88, 0.99) *	1.01 (0.98, 1.04)	1.00 (0.99, 1.02)	0.99 (0.97, 1.02)	1.02 (0.99, 1.04)
TG (mg/dL)		1.00 (0.99, 1.00)	1.00 (0.99, 1.01)	1.00 (0.99, 1.00)	1.00 (0.99, 1.00)	1.00 (0.99, 1.01)	1.00 (1.00, 1.01)
Homocysteine (μmol/L)		1.01 (0.97, 1.06)	1.01 (0.95, 1.08)	1.03 (0.97, 1.09)	1.00 (0.97, 1.04)	0.98 (0.94, 1.03)	1.02 (0.97, 1.07)
HbA1c (%, category)	≤5.6	Reference	Reference	Reference	Reference	Reference	Reference
	5.7–6.4	1.23 (0.71, 2.12)	0.51 (0.18, 1.41)	2.16 (1.04, 4.49) *	0.88 (0.63, 1.22)	1.09 (0.68, 1.76)	0.63 (0.39, 1.04)
	≥6.5	2.82 (1.33, 5.99) **	2.96 (0.85, 10.31)	2.96 (1.04, 8.39) *	1.23 (0.72, 2.12)	1.14 (0.54, 2.42)	1.25 (0.54, 2.91)

Statistically significant *p* values are indicated as *p < 0.05, **p < 0.01, ***p < 0.0001. MMSE, Mini-Mental State Examination; MoCA, Montreal Cognitive Assessment; BMI, body mass index; SBP, systolic blood pressure; DBP, diastolic blood pressure; HbA1c, glycosylated hemoglobin; FBG, fasting blood glucose; Hb, hemoglobin; TC, total cholesterol; HDL-C, high-density lipoprotein cholesterol; TG, triglycerides.

For women, after adjusting for covariates that may affect cognitive function, the risk of MMSE-measured cognitive impairment in the HbA1c 5.7–6.4% group was 2.16 times higher (95% CI, 1.04–4.49) than that in the normal HbA1c (≤5.6%) group, and the difference was significant. In the HbA1c ≥6.5% group, the risk of cognitive impairment was 2.96 times higher (95% CI, 1.04–8.39) than that in the normal HbA1c (≤5.6%) group, and the difference was significant. The model fit of the logistic regression analysis was tested using the Hosmer–Lemeshow test, which yielded a *p-*value of 0.602, indicating that the model was suitable. The ROC analysis results showed that MMSE could discriminate well between the cognitive impairment group and the normal cognition group in women (area under the ROC = 0.81, *p* < 0.0001).

## 4 Discussion

Cognitive impairment is a prominent feature of dementia; however, cognitive decline frequently occurs in older adults without dementia (39). In the community-based older population without dementia, the incidence of cognitive impairment assessed by the MMSE and MoCA was 12.5 and 44.3%, respectively. According to the MMSE evaluation, the HbA1c level was a risk factor for cognitive impairment in women.

HbA1c may be used as a predictor of fasting hyperglycemia and metabolic syndrome in Korean individuals without diabetes (40). A previous study (41) found a significant association between higher variability in HbA1c levels and cognitive decline in an older population without diabetes. The MMSE and MOCA are screening tests that are frequently used in clinical settings (42). In the Korea Genome Epidemiology Study Ansan cohort (2009–2010), cognitive function in older individuals was evaluated for the first time using the MMSE and MoCA cognitive function tools.

In this study, the HbA1c level was found to be a risk factor for MMSE decline in women. These results can be interpreted in the same context as reports (10) indicating a strong correlation between glycated hemoglobin and memory as well as the negative impact of chronically high blood sugar levels on cognition in a cohort of healthy older people without dementia or diabetes. Moreover, our results are well supported by those of a previous study showing that HbA1c levels were significantly inversely associated with cognitive performance. Furthermore, high HbA1c levels are correlated with notable reductions in fractional anisotropy after adjusting for covariates in healthy young adults (43). Previous studies (5, 9, 11, 44, 45) have shown relationships between elevated HbA1c levels and dementia-related outcomes, such as changes in hippocampal volume on neuroimaging or rates of cognitive decline. In older adults without neurological symptoms, brain volume loss accelerates with age, and the HbA1c level has been identified as a risk factor for increased brain atrophy rates (45). This means that high glucose levels may have harmful effects on the aging brain, such as cognitive decline or changes in hippocampal volume (9). In this study, fasting blood glucose and HbA1c levels were used as independent variables and indicators of blood glucose levels to investigate the relationship between HbA1c levels and cognitive impairment in older individuals without dementia. HbA1c (but not fasting glucose) was found to be a risk factor for cognitive impairment in community-dwelling older women when cognitive function was assessed using the MMSE. These findings provide evidence supporting the use of HbA1c as a marker for screening individuals with cognitive impairment.

Differences in the risk of dementia between men and women are well known, with women being at greater risk of developing cognitive decline than men (13). A previous study (11) identified an association between HbA1c levels and the risk of developing mild cognitive impairment or dementia in older women and showed that HbA1c was a predictor of cognitive decline, supporting our results. The risk of cognitive decline is greater in women than in men with mild cognitive impairment or Alzheimer’s disease, and the effect of the apolipoprotein ε4 allele is also greater in women (46–48). These data support our findings that HbA1c levels are associated with cognitive impairment in older individuals, with sex-dependent differences. Multiple logistic regression analysis of cognitive function (assessed using the MMSE), adjusted for covariates, showed that HbA1c significantly influenced cognitive impairment, whereas fasting blood glucose did not have a statistically significant effect.

In a previous study (49), patients with uncontrolled blood glucose levels had a 1.22-fold (95% CI, 1.10–1.34) higher risk of subjective cognitive decline than those with controlled blood glucose. This result is consistent with our findings, where the risk of cognitive impairment gradually increased with the increase in HbA1c levels: by 2.16 (95% CI, 1.04–4.49) and 2.96 (95% CI, 1.04–8.39) times for HbA1c 5.7–6.4% and HbA1c ≥6.5%, respectively. These results suggest that HbA1c is an important objective clinical indicator for identifying risk factors for cognitive impairment in older people.

When cognitive function was evaluated using the MMSE, diastolic blood pressure showed a significant effect on cognitive impairment in older people, consistent with previous findings (50–52). Our findings indicate that diastolic blood pressure is a significant risk factor for cognitive impairment in men, and future research exploring sex differences is needed. Our results based on MMSE and MoCA scores showed that the risk of cognitive impairment increased significantly with age and education level of elementary school or lower, regardless of sex. These findings are consistent with those of a previous study (53) that utilized the MMSE.

Concerns regarding cognitive decline due to population aging may be associated with cognitive dysfunction, which can be identified using neuropsychological and clinical tests (54). Considering that cognitive impairment can progress to mild cognitive impairment or dementia, timely and appropriate strategies are required in its early stages to reduce the individual and social burdens of the disease. The results of this study offer valuable insights for formulating strategies to promote successful aging and prevent sex-specific cognitive impairment among Korean older adults.

It is known that the MoCA is more sensitive to subtle cognitive impairment than the MMSE, but the use of the MoCA may decrease specificity (55). The reason why MoCA showed no association with HbA1c in the multiple logistic regression model analysis is explained as follows. First, Previous study (25) have shown that the MoCA is useful for the mild stages of the cognitive impairment spectrum (including MCI and mild AD), and the MMSE is superior for more advanced stages (AD patients with more significant functional impairment). One problem with MMSE is ceiling effect, which increases the likelihood that persons in predementia stages score within the normal range (≥24) (56). However, MoCA has less ceiling effect (56) and thus has high sensitivity for detecting mild cognitive impairment. Second, the cognitive domains and scores assessed by MoCA and MMSE are different (57). In this study, cognitive functions in different domains were assessed according to each test of MoCA and MMSE as presented in Appendix 1. MoCA includes items of visuospatial and executive function, and abstraction, and has fewer items of orientation in time and place than MMSE, making it difficult. Finally, the cognitive decline cutoff points of MoCA and MMSE are different. Depending on the cutoff point, the sensitivity and specificity of the test vary. The degree of influence of HbA1c varies depending on the sensitivity of reflecting the degree of decline in MoCA and MMSE scores. In this study, the MoCA and MMSE cutoffs (≤22 and ≤ 23, respectively) validated in Korea were used to determine cognitive decline. Therefore, it is possible that HbA1c is more relevant as a risk factor for cognitive decline at a later stage of cognitive impairment as suggested by the lack of association with the MoCA.

As clearly depicted in the scatterplot showing the correlation between MoCA and MMSE scores for the overall population in our study (Supplementary Appendix 3 Figure 1), there is overlap and cutoff in detecting cognitive impairment in both tests.

Our study had some limitations. First, establishing a causal relationship between HbA1c levels and cognitive impairment was challenging owing to the cross-sectional design of the study. Thus, only a simple association was presented. Second, HbA1c levels might not accurately reflect the true mean glycemia in conditions that affect red blood cell turnover, such as anemia, pregnancy, and end-stage renal disease (58). Third, administration of high doses of steroids (59–61) can lead to discrepancies between HbA1c results and the actual average blood glucose level. Finally, other limitations to the study could be the absence of apolipoprotein E(APOE) status, brain imaging data, or body fluid biomarkers [amyloid-*β* (Aβ) and tau protein] for Alzheimer’s disease (AD). Since preclinical AD could be a confounding factor as it is a major cause of cognitive decline in older adults.

However, this study had several strengths. To our knowledge, few studies have investigated the association between HbA1c levels (categorized as ≤5.6%, 5.7–6.4%, and ≥ 6.5%) and cognitive impairment in the older population. Our results suggest that elevated HbA1c levels may increase the risk of cognitive impairment in older women. From a clinical perspective, healthcare professionals should pay particular attention to older women with elevated HbA1c levels. Enhancing the management of glucose levels, as assessed by HbA1c, may help mitigate the risk of cognitive impairment in the older population. Future research investigating sex differences associated with cognitive impairment is warranted.

In conclusion, our analysis of cognitive function using the MMSE showed that the risk of cognitive impairment in women significantly increased when the HbA1c levels were above the normal range. This study is significant because it confirmed that HbA1c is a risk factor for cognitive impairment, going beyond its traditional role in assessing average blood glucose control and the risk of diabetes complications. Improving the control of glucose levels, as assessed by HbA1c levels, is recommended to mitigate the risk of cognitive impairment in older women.

## Data Availability

The KoGES data are publicly available at https://nih.go.kr/ and can be provided after evaluation of the research plan by the National Institute of Health, Korea Disease Control and Prevention Agency. The data used in this study were obtained from the Korean Genome and Epidemiology Study (KoGES; 4851-302), National Institute of Health, Korea Disease Control and Prevention Agency, Republic of Korea.
