# Supplementary material for: Association between elevated glycosylated hemoglobin and cognitive impairment in older Korean adults: 2009–2010 Ansan cohort of the Korean genome and epidemiology study
Source: Front Public Health. 2024 Nov 11;12:1417236. doi: 10.3389/fpubh.2024.1417236 (PMC11586203; doi:10.3389/fpubh.2024.1417236)
Supplement: Supplementary file 1 [file Data_Sheet_1.pdf]

Association between Elevated Glycosylated Hemoglobin and Cognitive Impairment in Older Korean Adults: 2009–2010 Ansan Cohort of the Korean Genome and Epidemiology Study

Supplementary Material

Appendix 1

Supplementary Table 1. Means and standard deviations of MMSE and MoCA domains

Domains	Points	Mean	SD	Scaled score
MMSE (N = 849)				
Orientation in time	5	4.88	0.44	97.6
Orientation in place	5	4.90	0.34	98.0
Registration	3	2.96	0.31	98.7
Attention and calculation	5	3.96	1.29	79.2
Recall	3	1.49	1.09	49.7
Oral and written language ability	8	7.31	0.98	91.4
Visuospatial function	1	0.84	0.37	84.0
Total	30	26.34	2.75	87.8
MoCA (N = 839)				
Visuospatial and executive function	5	3.78	1.24	75.6
Naming	3	2.70	0.62	90.0
Attention and calculation	6	4.92	1.31	82.0
Language	3	2.08	0.89	69.3
Abstraction	2	0.63	0.76	31.5
Delayed recall	5	2.06	1.54	41.2
Orientation in time and place	6	5.87	0.46	97.8
Total	30	22.37	4.11	74.6

MMSE, Mini-Mental State Examination; MoCA, Montreal Cognitive Assessment; SD, standard deviation

Appendix 2

Supplementary Table 2. Dispersion of continuous independent variables

Variables	N	Mean (SD)	Minimum	Q1	Median	Q3	Maximum	Range	IQR
Age (years)	853	66.4 (4.7)	59	62	66	70	77	18	8
BMI (kg/m^2)	852	24.8 (3.0)	16.1	22.9	24.7	26.6	34.8	18.7	3.7
SBP (mmHg)	846	120.0 (16.2)	80	110.0	119.0	130.0	212.0	132.0	20.0
DBP (mmHg)	846	74.6 (9.0)	50	68.0	74.0	80.0	108.0	58.0	12.0
Muscle mass (kg)	852	42.7 (7.5)	25.8	36.7	41.5	48.4	66.3	40.5	11.7
HbA1c (%)	853	5.9 (0.9)	3.7	5.5	5.7	6.1	12.0	8.3	0.6
FBG (mg/dL)	853	103.5 (31.1)	50	90.0	96.0	105.0	415.0	365.0	15.0
Insulin (µIU/mL)	853	10.6 (16.4)	1.0	6.4	8.3	11.1	349.0	348.0	4.7
Hemoglobin (g/dL)	853	13.7 (1.3)	8.1	12.7	13.6	14.6	17.7	9.6	1.9
TC (mg/dL)	853	197.6 (38.1)	79	171.0	197.0	222.0	337.0	258.0	51.0
HDL-C (mg/dL)	853	44.2 (10.9)	24	37.0	42.0	50.0	82.0	58.0	13.0
TG (mg/dL)	853	139.3 (74.3)	32	87.0	121.0	169.0	604.0	572.0	82.0
Homocysteine (µmol/L)	853	14.8 (5.3)	6.5	11.6	13.8	16.5	65.0	58.5	4.9
MMSE score	849	26.3 (2.8)	13	25.0	27.0	28.0	30.0	17.0	3.0
MoCA score	839	22.4 (4.1)	6	20.0	23.0	25.0	30.0	24.0	5.0

·Q1, 25th percentile; Q3, 75th percentile; IQR, interquartile range; MMSE, Mini-Mental State Examination; MoCA, Montreal Cognitive Assessment; BMI, body mass index; SBP, systolic blood pressure; DBP, diastolic blood pressure; HbA1c, hemoglobin A1c; FBG, fasting blood glucose; Hb, hemoglobin; TC, total cholesterol; HDL-C, high-density lipoprotein cholesterol; TG, triglycerides

Appendix 3

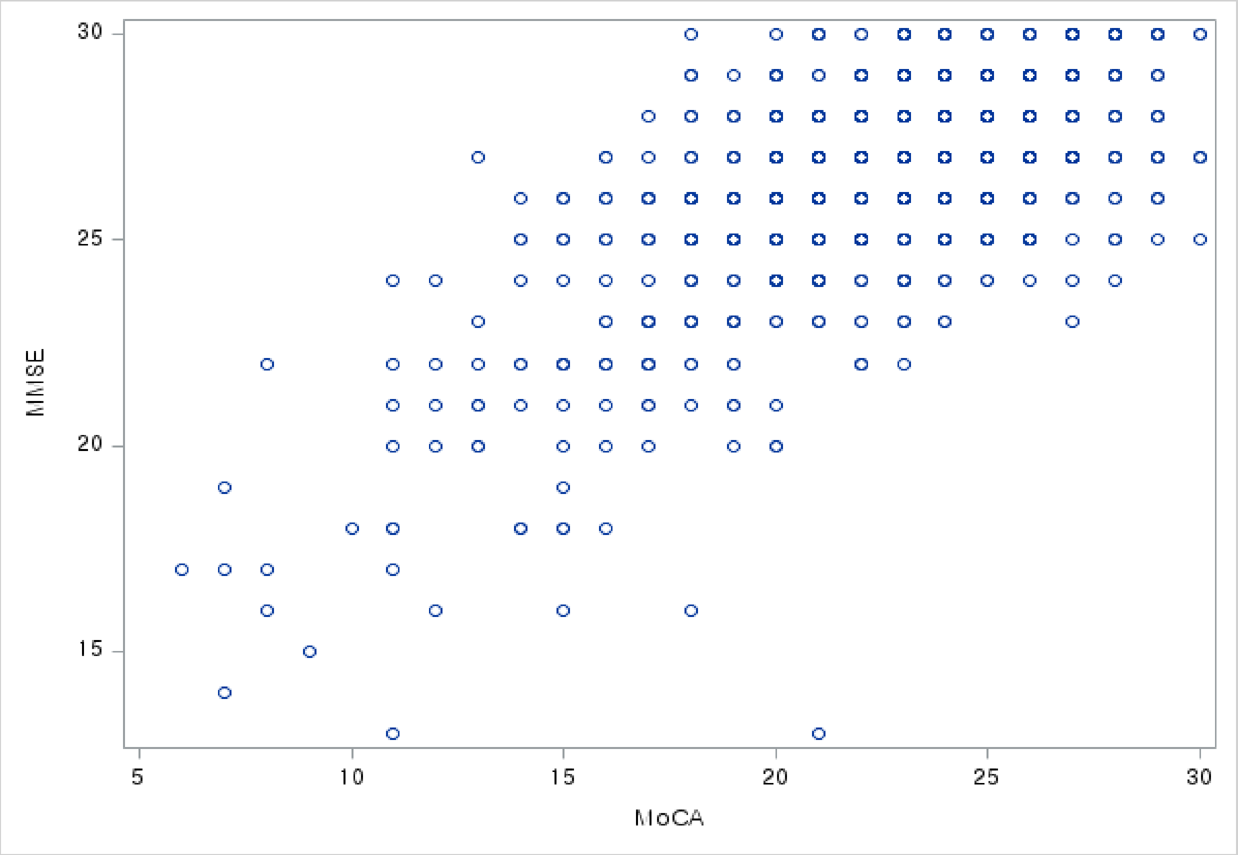

Supplementary Figure 1. The correlation between the Montreal Cognitive Assessment (MoCA) and Mini-Mental State Examination (MMSE) total scores in older Korean adults without dementia
